# The giant ciliate *Zoothamnium niveum* and its thiotrophic epibiont *Candidatus* Thiobios zoothamnicoli: a model system to study interspecies cooperation

**DOI:** 10.3389/fmicb.2014.00145

**Published:** 2014-04-07

**Authors:** Monika Bright, Salvador Espada-Hinojosa, Ilias Lagkouvardos, Jean-Marie Volland

**Affiliations:** ^1^Department of Limnology and Oceanography, University of ViennaVienna, Austria; ^2^Department of Microbiology and Ecosystem Science, University of ViennaVienna, Austria

**Keywords:** thiotrophic, sulfur-oxidizing, ciliate, symbiosis, mutualism, cooperation, wood fall

## Abstract

Symbioses between chemoautotrophic sulfur-oxidizing (thiotrophic) bacteria and protists or animals are among the most diverse and prevalent in the ocean. They are extremely difficult to maintain in aquaria and no thiotrophic symbiosis involving an animal host has ever been successfully cultivated. In contrast, we have cultivated the giant ciliate *Zoothamnium niveum* and its obligate ectosymbiont *Candidatus* Thiobios zoothamnicoli in small flow-through aquaria. This review provides an overview of the host and the symbiont and their phylogenetic relationships. We summarize our knowledge on the ecology, geographic distribution and life cycle of the host, on the vertical transmission of the symbiont, and on the cultivation of this symbiosis. We then discuss the benefits and costs involved in this cooperation compared with other thiotrophic symbioses and outline our view on the evolution and persistence of this byproduct mutualism.

## INTRODUCTION

**FIGURE 1:**
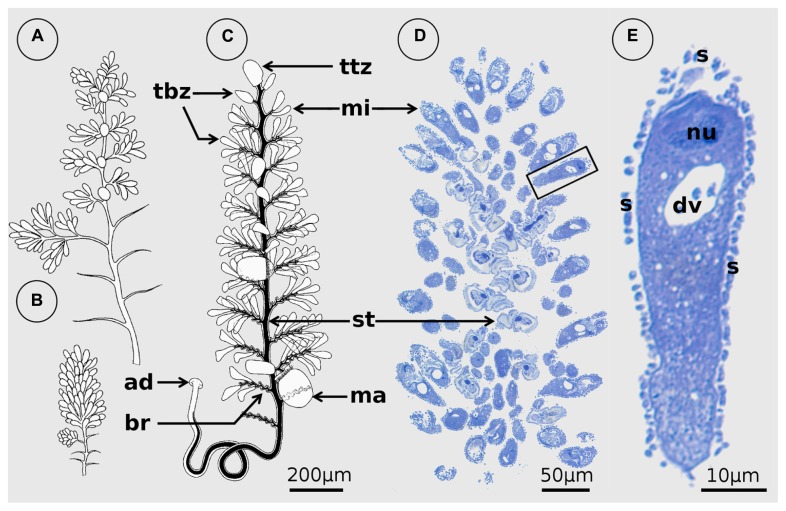
***Zoothamnium niveum.* (A,B)** Original illustrations modified from Hemprich and Ehrenberg (1829) showing the same colony expanded **(A)** and contracted **(B)**. **(C)** Drawing of a colony from the redescription of *Z. niveum* showing the different cell types: the macrozooid (ma), the microzooid (mi), the terminal branch zooids (tbz), and the terminal top zooid (ttz; modified from Bauer-Nebelsick et al., 1996a). **(D)** Microscopic observation of a longitudinal section of a *Z. niveum* colony. The stalk (st) of the contracted colony is visible as well as the numerous microzooids. **(E)** Detail of a single microzooid with macronucleus (nu) and digestive vacuole (dv), covered by its ectosymbionts (s).

The first illustration of a colonial ciliate from the Red Sea was published more than 180 years ago (Hemprich and Ehrenberg, 1829). Two years later, based on the small drawing of a single specimen, *Zoocladium niveum* was formally described and was named “small Abyssinian double bell” (Hemprich and Ehrenberg, 1831; translated by the first author; **Figure 1**). It was found on a rock at the coast of the Red Sea, probably close to the former kingdom of Abyssinia. Shortly thereafter, this species was placed in the earlier described genus *Zoothamnium* (Bory de Saint-Vincent, 1824). Ehrenberg (1838) observed in this specimen that “the whole stem suddenly contracted to a white knot” (p. 290; translated by the first author). Over the following decades, *Z. niveum* was discovered in other localities and with similar or slightly different morphology (see Bauer-Nebelsick et al., 1996a for further literature). Nonetheless, the typical white color, for which the species was named “niveum,” was not mentioned again until it was discovered by Jörg Ott in mangrove islands of Belize. Only then was it redescribed and its association with white, sulfide-oxidizing bacteria characterized (Bauer-Nebelsick et al., 1996a,b).

The white color in many sulfur-oxidizing (thiotrophic) bacteria is due to elemental sulfur inclusions, which are an intermediate product in the oxidation process of reduced sulfur species (Pflugfelder et al., 2005; Himmel et al., 2009; Maurin et al., 2010; Gruber-Vodicka et al., 2011). When involving animal or protist hosts, this type of association is termed thiotrophic symbiosis. Thiotrophic bacteria use hydrogen sulfide or other reduced sulfur species (see Childress and Girguis, 2011), which are typically produced biologically by anaerobic sulfate-reducing bacteria or geothermally at hydrothermal vents, to gain energy for carbon fixation (see Dubilier et al., 2008). Such bacteria, both free-living and host-associated, are extremely widespread at marine oxic–anoxic interfaces from shallow waters to the deep sea, including suboxic sediment layers, decaying plant matter, such as in sea grass meadows, mangrove peat, and wood, in whale bones, hydrocarbon seeps, and hydrothermal vents (Dubilier et al., 2008). Most symbioses are marine, but recently the first thiotrophic symbiosis was described from a freshwater limestone cave (Dattagupta et al., 2009). Thiotrophic symbionts belong to various clades of Gamma-, Epsilon- and, as recently discovered, also Alphaproteobacteria (Dubilier et al., 2008; Gruber-Vodicka et al., 2011).

The host taxa are even more diverse, although hydrogen sulfide is highly toxic (National Research Council, 1979) and eukaryotic hosts need to somehow cope with this poison. Extra- and intracellular endosymbioses as well as ectosymbioses are reported within six animal phyla (Nematoda, Platyhelminthes, Annelida, Arthropoda, Mollusca, Echinodermata) and one protist phylum (Ciliophora; see Ott et al., 2004; Stewart et al., 2005; Cavanaugh et al., 2006; Dubilier et al., 2008). All types of transmission modes – vertical from parents to offspring, horizontal from the environment, or mixed modes – are known within these prevalent bacterial symbioses in the sea (see Bright and Bulgheresi, 2010; Vrijenhoek, 2010).

Despite this dominance, research has been somewhat limited because many thiotrophic symbioses occur in poorly accessible, deep-sea environments. They are extremely difficult to maintain in the laboratory or even to culture. To our knowledge, only a few bivalves (for example, the lucinid *Codakia orbicularis*; Gros et al., 1997) were reared to maturity. This colonial ciliate, however, was successfully cultivated including the entire life cycle with the production of offsprings (Rinke et al., 2007). While bivalves exhibit intrinsically slower growth, and reproduction, the colonial ciliate has a much faster growth and reproduction, and a short life span. These characteristics along with easy access in shallow waters make this thiotrophic symbiosis of *Z. niveum* and its single bacterial partner, *Candidatus* Thiobios zoothamnicoli, a promising candidate for future studies. The present review summarizes our knowledge on this symbiosis and outlines our view on its evolution.

## THE HOST *Zoothamnium niveum*

*Zoothamnium niveum* belongs to a morphologically well-defined colonial ciliate genus of Peritrichida (Oligohymenophora) characterized by zooids that are connected by a common stalk. The contractile spasmoneme runs uninterrupted through the whole colony and bends in a “zigzag” pattern upon contraction (see Clamp and Williams, 2006). *Z. niveum* shares an alternate branching pattern with several other species such as *Z. alternans* Claparède and Lachmann 1858, but is much larger and has typical bell-shaped microzooids (Bauer-Nebelsick et al., 1996a; **Figure 1**). With a length of up to 1.5 cm it is by far the largest representative of this genus (Vopel et al., 2005).

**FIGURE 2:**
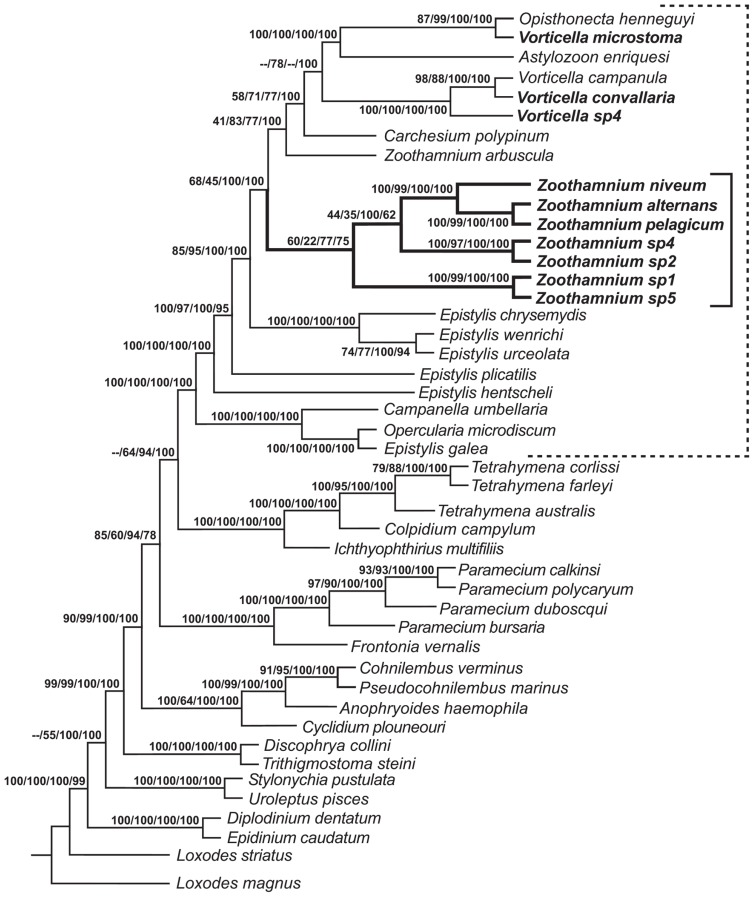
**Consensus tree formed from the four trees generated by phylogenetic analyses (Clamp and Williams, 2006).** Neighbor-joining (NJ) bootstrap value, maximum parsimony (MP) bootstrap value, maximum likelihood (ML) consensus value, and Bayesian consensus value are given as numbers on branches; missing values reflect minor differences in topology that could not be represented on the consensus tree. Solid bracket indicates species of *Zoothamnium*; dashed bracket indicates species of peritrichs. Species sequenced in Clamp and Williams (2006) are shown in bold type.

The 18S rRNA sequence from a population found on decaying mangrove leaves close to Fort Pierce, FL, USA and from a population collected from a whale bone in Tokyo Bay was almost identical, indicating an extremely wide geographic distribution (Clamp and Williams, 2006; Kawato et al., 2010). A sister taxa relationship of *Z. niveum* with *Z. alternans* + *Z. pelagicum* Du Plessis, 1891 was reported (Clamp and Williams, 2006; **Figure 2**). Both closely related species have been described with epibiotic bacteria (Dragesco, 1948; Fauré-Fremiet et al., 1963; Laval, 1968, 1970; Laval-Peuto and Rassoulzadegan, 1988). Epibionts of one morphotype consistently cover the pelagic *Z. pelagicum*. They were suggested to be cyanobacteria (Laval-Peuto and Rassoulzadegan, 1988). In *Z. alternans* it remains unclear whether the association is obligate for the host and involves a specific symbiont or merely represents unspecific microbial fouling.

**FIGURE 3:**
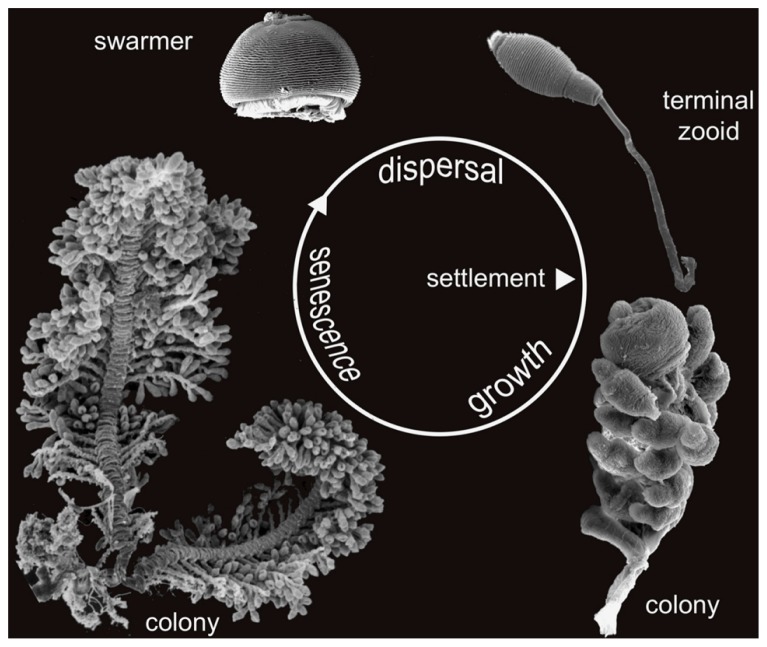
**Life cycle of *Zoothamnium niveum*.** Scanning electron microscopy of the different stages of development. The dispersive stage, the swarmer, is released from the colony and settles to grow a new colony. The new colony initially consists of a single cell, the terminal zooid, which divides to grow a whole colony. After a growing phase the adult colony enter a senescence stage. Not in scale.

The colonial host exhibits a central stalk with alternate branches and three cell morphotypes: terminal zooids on the tip of the stalk and each branch, feeding microzooids, and macrozooids (**Figure 1**). The latter develop on the base of the branches and leave the colony as swarmers to disperse and found new colonies (Bauer-Nebelsick et al., 1996a,b; **Figure 3**). Microzooids exhibit typical digestive structures with an oral ciliature and a cytopharynx (Bauer-Nebelsick et al., 1996b). Food vacuoles containing bacteria of similar size and microanatomical features as the symbionts are frequently found. The macrozooids, however, lack a cytopharynx, but their oral ciliature is fully developed. No food vacuoles were observed in macrozooids, leading to the conclusion that they are nourished by the microzooids (Bauer-Nebelsick et al., 1996b).

Sexual reproduction through conjugation has been described in some representatives of *Zoothamnium* (Furssenko, 1929; Summers, 1938), but never in *Z. niveum* (Bright M., personal observation). Asexual reproduction is through swarmers (Bauer-Nebelsick et al., 1996a,b). Macrozooid size varies considerably (20–150 μm). As soon as the somatic girdle (circular rows of cilia) is developed, macrozooids can leave the mother colony as swarmers. Somatic girdle development, however, is not correlated with macrozooid size (Bauer-Nebelsick et al., 1996a). The circumstances under which the somatic girdle develops prior to dispersal in the water column have not been studied.

**FIGURE 4:**
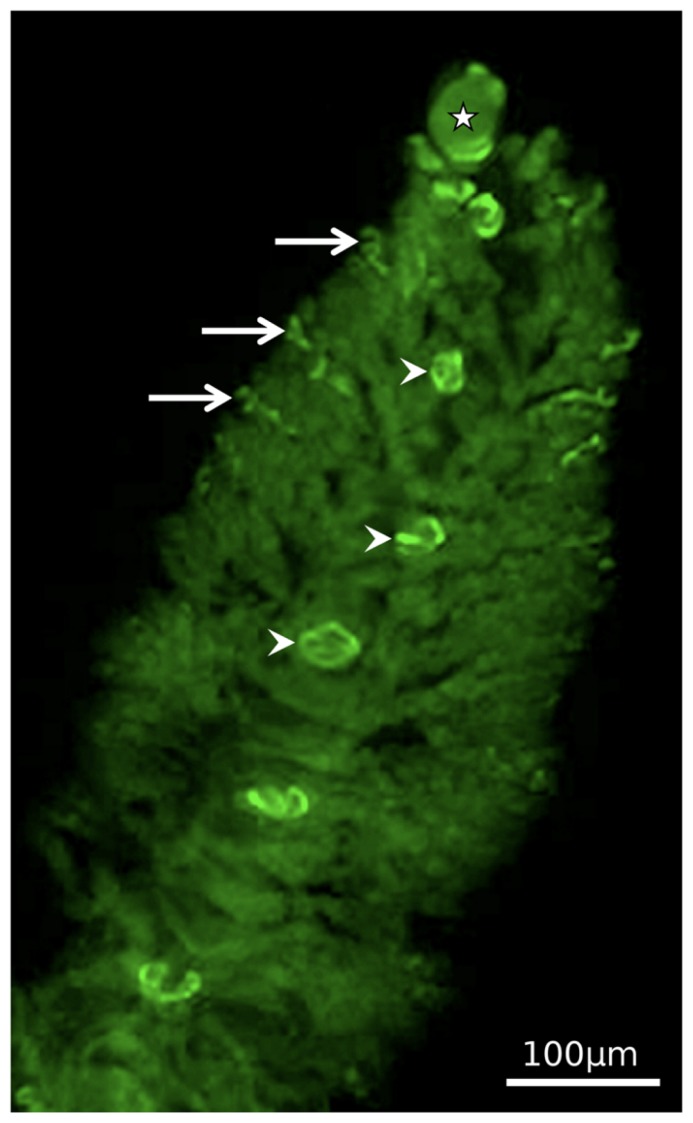
**General view of a *Zoothamnium niveum* colony showing the immunolocalization of BrdU incorporated into proliferating cells.** Labeled nucleus are observed in the terminal top zooid, some of the terminal branch zooids and in the macrozooids located along the stalk. Modified from Kloiber et al. (2009).

Using bromodeoxyuridine, a thymidine analog, and immunocytochemistry to study proliferation kinetics, Kloiber et al. (2009) corroborated that DNA synthesis is restricted to terminal zooids and macrozooids (**Figure 4**). The terminal zooid on the tip of the stalk produced the terminal zooids of each branch. Thus the number of branches is equivalent to the divisions of this top terminal zooid, and the youngest parts are on the tip of the colony, the oldest on the bottom. The division rate of the top terminal zooid decreased as the colony grew, but never ceased (Kloiber et al., 2009). The terminal zooids of the branches produced the microzooids. They had limited proliferation capacity, increasing the branch length with maximally 20 microzooids. At the base of the branches, macrozooids are produced. The number of macrozooids in large colonies with more than 50 branches was greater (about 15) than in small colonies with less then 50 branches (about 6). In macrozooids, DNA synthesis occurred on branches, but the cell cycle was arrested until swarmers left the colony. They probably resume mitosis and cell division upon settlement, when they in fact become the top terminal zooid (Kloiber et al., 2009).

## THE SYMBIONT *Candidatus* Thiobios zoothamnicoli

**FIGURE 5:**
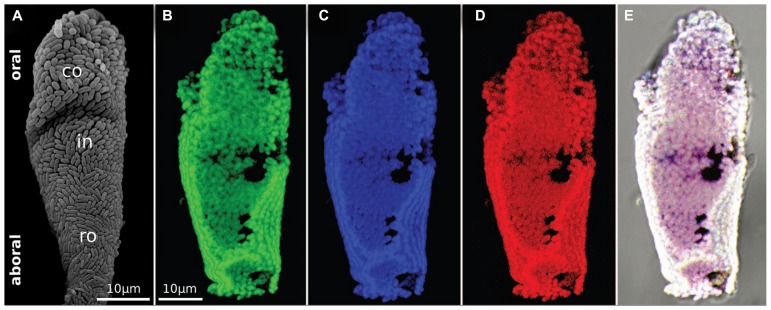
**The monospecific ectosymbiont monolayer.** **(A)** SEM observation of a microzooid showing the monolayer of bacteria covering the host cell. The two morphotypes are visible, rod-shaped symbionts at the aboral part and coccioid symbionts at the oral part. **(B–D)** FISH micrographs of a single microzooid after hybridization with a general bacterial probe in green **(B)**, a gammaproteobacteria specific probe in blue **(C)**, and a *Cand.* Thiobios zoothamnicoli specific probe in red **(D)**. **(E)** Overlay of the three previous micrographs (Rinke et al., 2006).

A single 16S rRNA phylotype covers the host in a strict monolayer, except for the most basal part of the colony (Rinke et al., 2006; **Figure 5**). Depending on the location of the host, this phylotype grows either as rod or as cocci. They are rods on the stalk, branches, terminal zooids, macrozooids, and on the aboral parts of microzooids. The oral part of the microzooids, is covered with cocci, with a gradual change from cocci to rods from the oral to aboral side. The most basal, senescent parts of the colony are overgrown with all kinds of microbes and the symbiont is partly lost (Bauer-Nebelsick et al., 1996a,b; Rinke et al., 2006).

The symbionts have a cytoplasmic and an outer cell membrane, typical of Gram-negative bacteria (Bauer-Nebelsick et al., 1996b). Raman microspectroscopy revealed vesicles filled with S_8_ sulfur (Maurin et al., 2010). Experiments in Cartesian divers showed a rapid decrease of oxygen consumption within 4 h, which remained at a low level for 24 h under normoxic conditions. This suggests that elemental sulfur is used with oxygen as an electron acceptor for about 4 h, during which the colonies are depleted of this intermediate storage product and turn pale. The baseline of oxygen consumption represents the respiration of host and symbiont. After injecting 100 μmol L^-1^ΣH_2_S (sum of H_2_S, HS^-^, S^2-^), oxygen consumption was increased and rapidly decreased again. This suggests that the sulfide pulse enables the symbionts to briefly resume their chemoautotrophic activity (Ott et al., 1998).

Each host population associates with a single specific symbiont (based on 16S rRNA). The symbiont from Twin Cays, Belize, was tentatively named *Cand.* Thiobios zoothamnicoli (Rinke et al., 2006). The similarity between this and another population from Calvi, Corsica, was 99.7% (Rinke et al., 2009) and 99.2% to a Pacific population, termed “ectosymbiont of *Zoothamnium niveum*” (Kawato et al., 2010). The internal transcribed spacer (ITS) was also highly similar between the Twin Cays and Calvi population (Rinke et al., 2009). Genes for the key enzyme in the Calvin Benson cycle for carbon fixation (ribulose 1,5-bisphosphate carboxylase/oxygenase) and for sulfur metabolism (APS reductase, dissimilatory sulfite reductase) were discovered (Rinke et al., 2009).

Besides other strains of *Cand.* Thiobios zoothamnicoli recovered from different *Z. niveum* isolates, the closest relatives, as revealed by 16S rRNA gene phylogenetic analysis, of *Cand.* Thiobios zoothamnicoli all belong to a well separated group of uncultivated sulfur oxidizing bacteria related to gamma proteobacteria (Rinke et al., 2006, 2009; Kawato et al., 2010).

**FIGURE 6:**
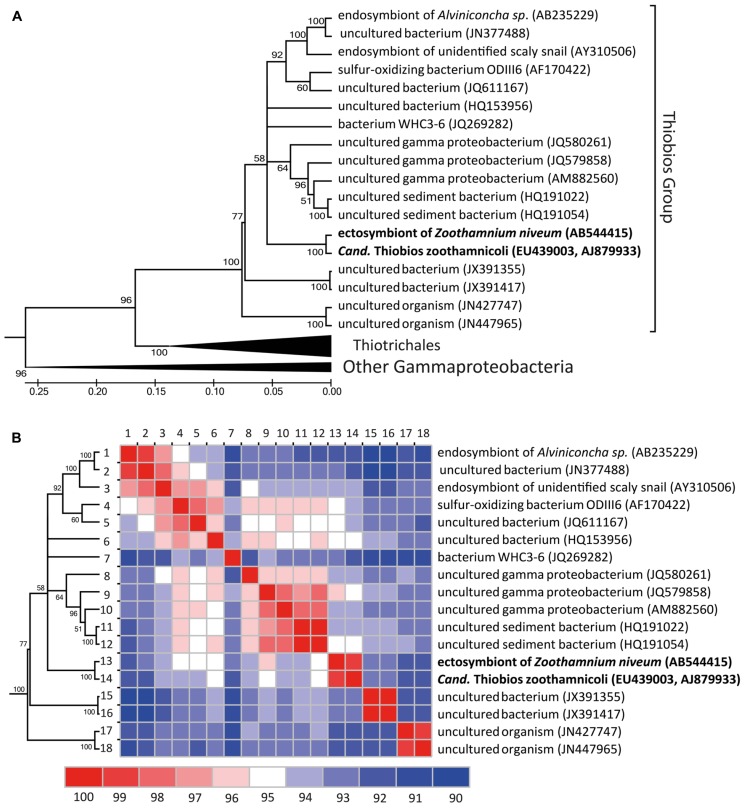
**Phylogenetic diversification of the *Cand.*** Thiobios zoothamnicoli neighborhood. **(A)** Maximum likelihood phylogenetic tree (GTR model, 1000 bootstraps) of all long (>1300 nt), with good pintail value (>60) and non-redundant 16 rRNA sequences similar to *Cand.* Thiobios zoothamnicoli available in the SILVA database (Quast et al., 2013). The tree with the highest log likelihood is shown and is drawn to scale, with branch lengths measured in number of substitutions per site. Evolutionary analyses were conducted in MEGA5 (Tamura et al., 2011). **(B)** Similarity matrix of the 16S rRNA sequences belonging to the *Cand.* Thiobios zoothamnicoli group. The similarity was calculated as the percentage of identical positions over all shared positions (not considering gaps) for each pair of sequences in the multiple sequence alignment and visualized using JColorGrid (Joachimiak et al., 2006).

The updated phylogenetic analysis reveals a group currently 19 16S rRNA sequences (all current close relatives in public databases; **Figure 6**). Overall this Thiobios group is dominated by free-living bacteria of shallow-water environments of all temperate to tropical oceans. Analyses restricted to the 16S rRNA gene provides insufficient resolution to fully clarify the evolutionary relations among the available representatives populating this branch of the tree, a problem that can only be resolved with genomic sequencing of targeted members. Nevertheless, symbiosis apparently evolved twice in the shallow waters as ectosymbioses in the Thiobios group: in *Z. niveum* and in the archaea *Giganthauma karukerense* (Muller et al., 2010). The available fragment of 16S rRNA from this archaea has a similarity of 93% to *Cand.* Thiobios zoothamnicoli (note that this sequence fragment is not included in **Figure 6**). In addition another clade of the Thiobios group colonized shallow-water and deep-sea vents, whereby endosymbiosis with two different gastropod hosts evolved.

## HABITAT AND ECOLOGY

**FIGURE 7:**
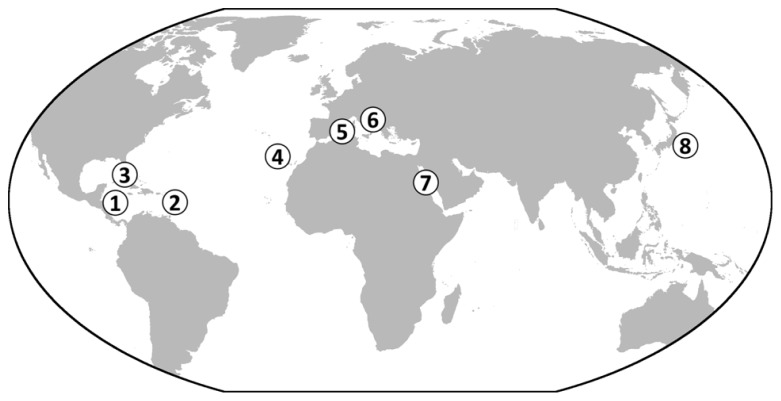
**World map showing the known occurrences of *Zoothamnium niveum*.** So far, colonies of the ciliate have been found in the Caribbean on mangrove peat wall, sunken wood and leaf debris (**1**, Twin Cays Island, Belize; **2**, Guadeloupe, French West Indies; Rinke et al., 2006; Laurent et al., 2009, 2013). In the Gulf of Mexico, the symbiosis was found in the Florida Keys **(3)** (Bauer-Nebelsick et al., 1996a). In the Atlantic Ocean, it was found in Lanzarote in the Canary Islands **(4)** (Wirtz and Debelius, 2003). It was also collected from rocks near sea grass debris accumulation in the Mediterranean Sea (**5**, Corsica, France) and in the Adriatic Sea from sunken wood **(6)** (Bright M., personal observation). The original description reported *Z. niveum* from the Red Sea **(7)**, and recently it has been described growing on bones of a whale fall deployed in the Tokyo Bay, Japan **(8)** (Kawato et al., 2010).

The data increasingly point to a widespread occurrence of the giant ciliate symbiosis on or near decaying organic material in shallow tropical to temperate waters. So far, this symbiosis has been detected in the biogeographic provinces of the Caribbean Sea (Bauer-Nebelsick et al., 1996a; Clamp and Williams, 2006; Laurent et al., 2009), the Atlantic Ocean (Clamp and Williams, 2006; Wirtz, 2008), the Mediterranean Sea (Rinke et al., 2007; Wirtz, 2008), the Red Sea (Ehrenberg, 1838), and the Pacific Ocean (Kawato et al., 2010; **Figure 7**).

**FIGURE 8:**
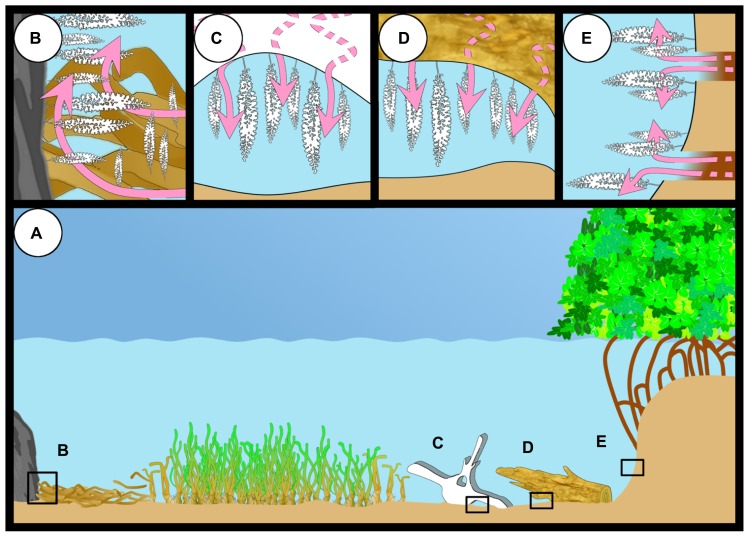
**The different habitats of *Zoothamnium niveum* (A).** The giant ciliate can colonize hard substrate close to sea grass debris accumulation where sulfide (pink arrows) is produced or grow directly on the sea grass debris itself **(B)**. They have also been reported from a whale bone recovered from the deep sea and experimentally deployed in shallow waters **(C)**, from sunken wood **(D)**, and mangrove peat walls where degrading vegetal debris including rootlets **(E)**.

In tropical and subtropical regions, the giant ciliate colonizes mangrove peat (mainly composed of wood; Lovelock et al., 2011) and sunken wood and leaves of the mangrove Rhizophora mangle (Bauer-Nebelsick et al., 1996a; Clamp and Williams, 2006; Laurent et al., 2009). In temperate waters, this ciliate inhabits whale falls (Kawato et al., 2010), wood (Bright M., personal observation), and sea grass debris of *Posidonia oceanica* (Rinke et al., 2007; Wirtz, 2008; **Figure 8**).

The current findings are all restricted to shallow subtidal waters, but the depth limits remain to be investigated. Mangrove trees occur in the intertidal, and sea grasses are limited to the euphotic zone. Wood may be transported into the deep sea and potentially could be colonized by this symbiosis. A sperm whale bone, recovered from about 1000 m depth in Sagami Bay without this symbiosis, was colonized by *Z. niveum* after the bone was deployed in 5 m depth in Tokyo Bay for 1 year (Kawato et al., 2010).

Detailed studies on colony distribution on peat walls were conducted at the mangrove island Twin Cays, Belize (Ott et al., 1998). On average, 1200 giant ciliate colonies m^-2^ were found between 30 cm below low water level down to the lower end of the peat wall at about 2 m depth. They were patchily distributed in groups of 26 ± 17 colonies, with maxima of more than 100 colonies per patch. Interestingly, many colonies thrive in areas where the microbial mat of the peat surface was disturbed, e.g., after decomposed rootlets fall out. Such conduits were suggested to be analogous to hydrothermal vents, where vent fluid emerges from the basalt and mixes with the oxygenated overlain seawater (Ott et al., 1998).

**FIGURE 9:**
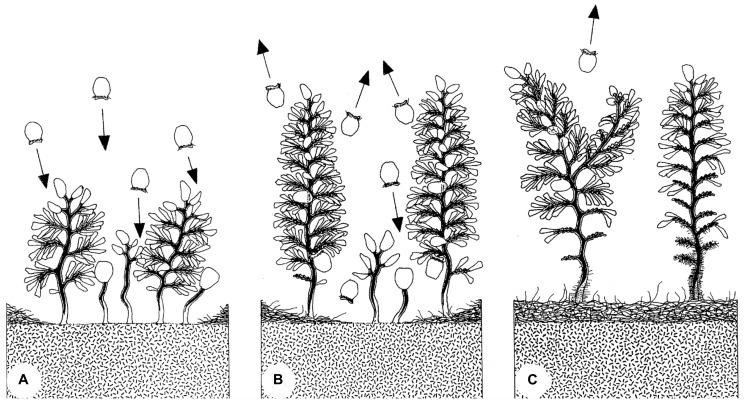
**Evolution of a patch of *Zoothamnium niveum* colony.** The swarmers colonize a disturbed area **(A)**. The settled colonies grow and start releasing new swarmers during a maturation phase **(B)**. Finally, the colonies enter a senescent phase **(C)**. Mature colonies are losing microzooids at the bottom part of the stalk, which starts to be overgrown by a variety of bacteria (Ott et al., 1998).

Colonization and succession of artificially disturbed surfaces on mangrove peat led to the distinction of initial patches with small colonies, followed by mature patches with colonies of all sizes, and senescent patches with large colonies. The latter were characterized by loss of zooids on the lower branches and were often overgrown by other microbes on the lower colony. A life expectancy of about 3 weeks was estimated based on the disappearance of such colony groups (Ott et al., 1998; **Figure 9**).

The microhabitat of *Z. niveum* is temporarily highly dynamic in terms of sulfide and oxygen concentrations. Measurements of oxygen and sulfide on peat surfaces from Twin Cays (Belize) were conducted in the lab (Ott et al., 1998; Vopel et al., 2001, 2002) and *in situ* (Vopel et al., 2005). Further *in situ* measurements of wood surfaces colonized by ciliates from Guadeloupe were carried out (Laurent et al., 2009). Adjoining areas of peat or wood devoid of ciliates always exhibited different oxygen and sulfide concentrations (Ott et al., 1998; Vopel et al., 2001, 2002; Maurin et al., 2010), suggesting a highly specific chemical environment *Z. niveum* inhabits.

Large-scale sulfide fluctuations were associated with the tidal cycle. The highest maxima were recorded at high tide, the lowest at low tide (Laurent et al., 2009). Small-scale fluctuations of sulfide and oxygen at the opening of conduits on peat walls were caused by pulse exchange between deoxygenated, sulfidic seawater in conduits and oxygenated seawater adjacent to peat surface (Vopel et al., 2005). Peak values occurred in periods of 10–100 s. Depending on flow speed, sulfide was high or low (Vopel et al., 2005). Ciliates preferentially settled in areas of about 250–300 μmol L^-1^ΣH_2_S and oxygen values of about 20 μmol L^-1^ (Ott et al., 1998; Vopel et al., 2005). In contrast, the wood surface colonized by the ciliates had only about 100 μmol L^-1^ΣH_2_S. Fluctuations between these maxima and almost fully oxygenated seawater occurred in less than one hour (Laurent et al., 2009).

**FIGURE 10:**
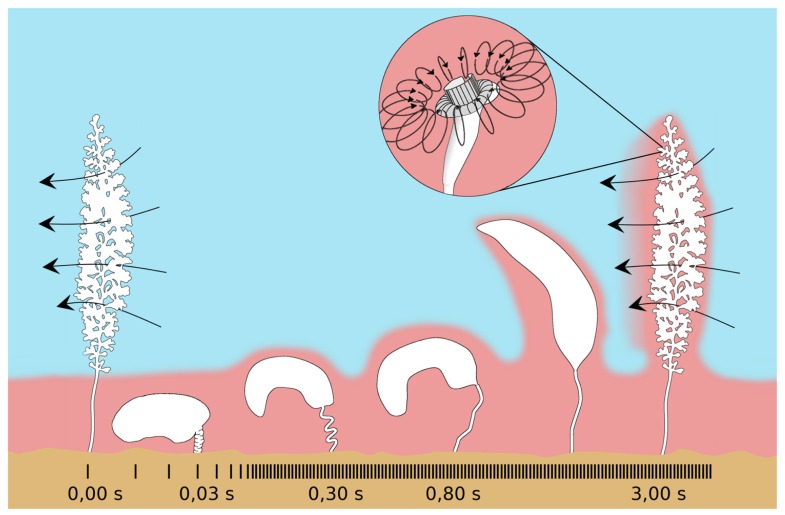
**Schematic drawing of the contraction pattern of *Zoothamnium niveum*.** The fast contraction (520 mm s^-1^) brings the colony in the sulfidic boundary layer (pink), then a slow extension movement bring it back to the oxygenated water (blue) dragging along sulfide from the boundary layer. Once extended, the cilia from the microzooids (insert) start beating again creating a toroidal vortex around the upper part of the cell (curved arrows). This current allows the cell to filter the surrounding water to gain food and, as a side effect, it also mixes the sulfide and the oxygen allowing the ectosymbionts to access both the electron donor and the electron acceptor. The beating of the cilia from all the cells of the colony also creates a general current perpendicular to the long axis of the colony (long arrows). Modified from Vopel et al. (2002).

In addition, the host’s peculiar behavior of contracting and expanding, along with currents generated by the feeding microzooids, change the chemical environment (**Figure 10**). Colony contractions are extremely fast (520 mm s^-1^) and occur on average every 1.7 min. The zooids bunch together and the colony whips downward toward the peat surface followed by slow expansions, which are about 700–1000 times slower than contraction (Vopel et al., 2002). During slow expansion, sulfidic water sticks to the colony and is dragged along upward (Vopel et al., 2001). After fully expanded, the microzooids resume filter feeding by beating their oral cilia (Vopel et al., 2002). The Reynolds numbers change from about 102 during contraction to 10^-1^ during expansion (Vopel et al., 2002), and the symbionts may overcome the diffusion-limited substrate supply by beating of host cilia (Vopel et al., 2005).

## TRANSMISSION

Transmission is vertical: the macrozooids that leave the mother colony to build new colonies are also covered with the symbionts (Bauer-Nebelsick et al., 1996a,b). This has been confirmed with two symbiont-specific 16S rRNA probes (ZNS 196, ZNS 1439; Rinke et al., 2006) for both the population in Twin Cays (Belize) and in Calvi (Mediterranean Sea; Rinke et al., 2006, 2009). For the whale fall populations, one of the specific probes (ZNS 196) was tested and proved positive (Kawato et al., 2010).

This model system might be especially interesting from an evolutionary point of view. In general, horizontal transmission, in which the symbiont is picked up from the environment each generation anew, is considered to be the ancestral mode of symbiont transfer between generations. Mixed modes or strict vertical transmissions are assumed to have evolved later (Bright and Bulgheresi, 2010). In contrast, in the *Z. niveum* symbiosis, we suggest that vertical transmission is the ancestral mode of transmission. This interpretation is based on the simple design of an ectosymbiotic partner covering also the asexually produced propagules.

Vertical transmission, however, may not be the only option. The symbiont’s location on the host surface potentially allows for symbiont replacement by other bacteria from the surrounding environment. Moreover, release of symbionts due to sloppy feeding by the host and/or upon host death may support a free-living population from which the symbiont population could be re-inoculated. In contrast, strictly vertically transmitted symbionts no longer occur in the free-living environment and have co-evolved with their hosts (Bright and Bulgheresi, 2010). Thus, the potential of additional horizontal transmission in this model system should be explored in the future: it would influence the dynamics and demography of the symbiont population dramatically (see Vrijenhoek, 2010).

## CULTIVATION OF SYMBIOSIS

Instead of experimentally creating a sulfide and oxygen gradient as found in nature, the symbiosis was successfully cultivated with populations from Calvi in a flow-through respirometer system with stable conditions (Rinke et al., 2007). The continuous flow of all chemicals enables breaking the host’s control over the access to these chemicals and therefore also manipulating the environmental conditions for both partners. Optimal conditions (24–25°C, salinity 40, pH 8.2, ~ 200 μmol L^-1^ O_2_, 3–33 μmol L^-1^ΣH_2_S, flow rate 100 ml h^-1^) yielded a 10-fold increase in host colonies in 1 week. The mean life span of each colony was 11 days and host division rates of the top terminal zooid ranged from 4.1 to 8.2 day^-1^ during the first 8 days of growth phase; this was followed by a senescence phase during which more microzooids on branches were dying than being produced (**Figure 3**). In contrast, with no external sulfide source under normoxic conditions, growth was slower and the life span was considerably reduced to about 4 days (Rinke et al., 2007).

As expected due to uniform environmental conditions in this steady flow system, also uniform, rod-shaped symbionts covered the entire host. This finding supports the hypothesis that the ciliary beating in microzooids highly influences symbiont performance (Vopel et al., 2005). Furthermore, the frequency of dividing symbionts was taken as a measure of fitness. On the upper parts of microzooids, fitness was higher under optimal cultivation conditions compared to the *in situ* population. Fitness on the lower part of the microzooids was similar between the two populations.

Comparing the cultures of the Mediterranean and Caribbean populations, the latter reached maximal size within 4 days and had a mean life span of 7 days (Ott and Bright, 2004; Ott et al., 2004). The average water temperature of the former culture was 24–25°C, of the latter about 28°C (Vopel et al., 2001). The observed differences might reflect elevated metabolic rates under warmer conditions, leading to faster growth and shorter life span compared to colder conditions (Rinke et al., 2007).

## BENEFITS AND COSTS

The question of benefits for both partners, which should exceed the costs in mutualism, is difficult to answer. It requires comparisons between host and symbiont fitness of free-living cultures as well as of cultures in which the partners cooperate or defect (Buston and Balshine, 2007). Appropriate experiments have proven extremely difficult to carry out. In thiotrophic symbioses, several lines of thought have been pursued, but direct evidence is scarce. Several potential benefits have been investigated for the host, including direct nourishment by the symbiont as well as detoxification of sulfide, and for the symbiont, including the provision of substrates for sulfur oxidation and carbon fixation and a competition-free habitat (see Fisher and Childress, 1992; Ott et al., 2004; Stewart et al., 2005; Cavanaugh et al., 2006; Dubilier et al., 2008).

In several systems, nourishment of the host at some costs to the symbiont has been shown. Fast release of fixed organic carbon and digestion of symbionts are the two means of translocation from the symbiont to the host, for example, in the vestimentiferan tubeworm *Riftia pachyptila* (Felbeck, 1985; Felbeck and Jarchow, 1998; Bright et al., 2000) and the bivalves *Loripes lucinalis*, *Lucinoma aequizonata*, and *Solemya reidi* (Felbeck, 1983; Fisher and Childress, 1986; Distel and Felbeck, 1988; Herry et al., 1989). Also, preliminary studies on *Z. niveum* and *Cand.* Thiobios zoothamnicoli point to both translocation processes using ^14^C bicarbonate pulse chase incubations and tissue autoradiography (Rinke, 2002). After short pulses of 15 min label was present over host tissue indicating release, and after long pulses of 3 h and chases of 12 and 24 h, respectively, this label increased indicating digestion (Rinke, 2002). In addition, food vacuoles contained bacteria with the same size and shape as the symbiont with its typical sulfur vesicles (Bauer-Nebelsick et al., 1996b).

In some thiotrophic symbioses the digestive system is completely reduced, for example, in siboglinid tubeworms and gutless oligochaetes (see Dubilier et al., 2008). Here, the entire food should come from the symbiont. In other systems the digestive system still functions, additionally allowing for “normal” feeding. The microzooids in *Z. niveum* also have a functioning digestive system (Bauer-Nebelsick et al., 1996a,b). The degree to which host nourishment depends on symbionts or ingested prey has not been studied in any system yet. However, cultivation experiments in *Z. niveum* show that host fitness (measured as host growth and life span) was considerably decreased when symbionts were forced to defect. *Cand.* Thiobios zoothamnicoli could not fix carbon under normoxic culture conditions without sulfide (Rinke et al., 2007). The only means of nourishment left for the host were symbiont digestion and food uptake from the surrounding seawater. This indicates that a considerable portion of food comes from the symbionts.

Sulfide is highly toxic to aerobic eukaryotes (National Research Council, 1979). It inhibits cytochrome *c* oxidase, the eukaryote terminal enzyme of the mitochondrial electron transport chain (Dorman et al., 2002). Accordingly, the hosts of thiotrophic symbionts are challenged in providing their symbionts with sulfide while at the same time avoiding poisoning. Detoxification of sulfide through uptake and oxidation by symbionts has been proposed several times (Somero et al., 1989). Short incubations with Na_2_^35^S and autoradiographic analysis in the stilbonematid *Eubostrichus dianae* showed that most uptake was in the thiotrophic ectosymbionts (Powell et al., 1979). Future studies are urgently needed using aposymbiotic hosts exposed to sulfide in order to determine whether symbiont presence (with their sulfide oxidation capabilities) affects host fitness.

Access to oxygen and sulfide for thiotrophic ectosymbionts is generally facilitated by the host’s behavior (Ott et al., 2004). Migrations through the chemocline in sediments have been reported in the ciliate *Kentrophoros* ssp. (Fenchel and Finlay, 1989), the stilbonematin nematodes (Ott et al., 1991) and the gutless oligochaetes (Giere, 1992). Polz et al. (1999, 2000) observed the shrimp *Rimicaris exoculata* swimming in and out of hydrothermal vent fluid as well as ventilation of the chamber in which its symbionts reside. In *Z. niveum*, the host contracts and expands continuously, facilitating switches between sulfidic and oxygenated seawater (Ott et al., 1998). The symbionts on the host’s surface were suggested to overcome the diffusion limitations of their substrate supply by two processes: feeding currents generated by the host, and the pulsed advection of sulfidic seawater from the peat caused by interactions of the boundary layer flow with groups of ciliates (Vopel et al., 2005). Interestingly, all the symbionts exposed to the feeding currents are larger and coccoid in shape, while the symbionts on the other host part are less favored and thus remain smaller and rod-shaped (Rinke et al., 2007). This emphasizes the importance of host-generated ciliary currents.

Although *Cand.* Thiobios zoothamnicoli is tightly associated with its ciliate host, the ectosymbiotic location does not provide shelter from competing microbes. Nevertheless, most parts of the host are exclusively covered by the symbiont, pointing to mechanisms developed against unspecific colonization. Microbial fouling on more basal, older host parts suggests that the host controls colonizers until it become senescent. Only then do other microbes appear on top of the symbionts, sometimes replacing them (Bauer-Nebelsick et al., 1996b).

Detailed analyses elucidated the importance of the host surface for colonization and of host behavior for the symbiont population density (Røy et al., 2009). Sulfide transport and estimated oxygen consumption were incorporated in a model of sulfide requirements sustaining chemoautotrophic growth by analyzing the flow field around individual zooids. Fluxes of 6.61 μmol O_2_ m^-2^ s^-1^ and 3.19 μmol ΣH_2_S m^-2^ s^-1^ were calculated. This model suggests that sulfide uptake rates are 100 times larger for host-associated symbionts than for free-living bacteria on flat surfaces (Røy et al., 2009).

Some evidence points to mutualism in this ciliate symbiosis. While the host benefits from the symbiont’s organic carbon, translocated to the host (Rinke, 2002), the host’s costs to carry an ectosymbiotic coat during all life stages have not been explored. Especially the costs involved in transporting the symbiont during dispersal in the water column are unknown (Genkai-Kato and Yamamura, 1999). Swarmers might move in the boundary layer close to the peat surface, enabling uninterrupted thiotrophic symbiont functioning. Alternatively, they might migrate through the oxygenated water column and, depending on dispersal time, must deal with a non-functioning symbiont; this would potentially incur some costs to the host. Overall, the host is by far the largest representative in the genus *Zoothamnium* (see Bauer-Nebelsick et al., 1996a), indicating that benefits exceed costs.

The symbiont benefits from the host, which provides large surfaces or colonization and therefore supports enhanced symbiont population density with optimal conditions for sulfide oxidation and carbon fixation compared to flat surfaces (Røy et al., 2009). This colonization appears to be host controlled: space is allocated exclusively to the symbiont, enhancing symbiont fitness. The symbiont’s costs involve population reduction through digestion and possible host controlled enhanced leaking of fixed carbon to the host as has been shown for the photoautotrophic *Symbiodinium* in corals (see Trench, 1979). Such leaking processes occur to a certain degree in free-living microalgae and autotrophic bacteria, but are enhanced when the microalgae are host associated (see Trench, 1979). Also the thiotrophic endosymbiont of the giant tubeworm *Riftia pachyptila* leaks organic carbon when artificially separated from its host (Felbeck and Jarchow, 1998). Whether the ciliate host enhances this naturally occurring leaking process remains to be studied.

## EVOLUTION AND PERSISTENCE

A longstanding paradigm in cooperation theory depicts the evolution of mutualism from parasitism (Roughgarden, 1975; Ewald, 1987; Lipsitch et al., 1996; Yamamura, 1996). It has been argued that through vertical transmission lower virulence is selected and thus shifts the relationship toward a beneficial one. More recently, this general hypothesis has been rejected because in many systems phylogenetic information suggests mutualism can also evolve *de novo* from previously free-living partners or from previous mutualistic associations (Douglas, 2010; Sachs et al., 2011). *De novo* evolution of the *Cand.* Thiobios zoothamnicoli – *Z. niveum* mutualism is also the most likely scenario. Based on 16S rRNA, *Cand.* Thiobios zoothamnicoli is most closely related to a variety of free-living bacteria (**Figure 6**). Furthermore, no pathogens or parasites are known with sulfur-oxidizing, autotrophic metabolism (Dubilier et al., 2008).

A mathematical model predicts that vertical transmission can evolve when the costs for vertical transmission are low, the availability of free-living symbiont is poor, and byproduct usage is high on both sides (Yamamura, 1993, 1996; Genkai-Kato and Yamamura, 1999). While we cannot comment on the first two parameters in the giant ciliate symbiosis, there are some indications that byproduct usage is present and played an important role for the evolution of this vertically transmitted symbiosis.

Byproduct benefits involve one partner providing goods to the other at no costs, but rather as an automatic, coincident consequence of selfish traits (West-Eberhard, 1975; Connor, 1986, 1995; Hauert et al., 2006). Such byproduct benefits are considered to be important in the initiation of mutualism (Sachs et al., 2011). This self-interest action benefits both the actor and the associated recipient. Byproduct benefits, however, do not challenge evolutionary theory because both partners cooperating is favored over one partner cooperating and the other one defecting (Hauert et al., 2006) and have been largely neglected (Douglas, 2010).

**FIGURE 11:**
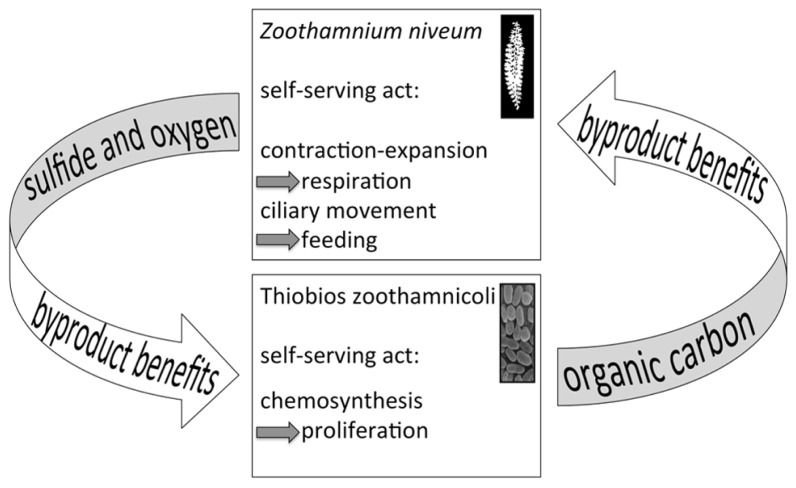
**Diagram of the putative byproduct mutualism.** The host’s behaviors to contract and expand and the ciliary movement are self-serving acts to gain access to oxygen for respiration and for feeding, respectively. As a byproduct, sulfide and oxygen is provided to the symbiont. On the other hand, the symbiont fixes carbon as a self-serving act to grow and as a byproduct nourishes the host.

Several characteristics of the present symbiosis may point to byproduct benefits, one provided by the symbiont to the host, the other provided by the host to the symbiont – at no costs. The leaking of fixed carbon from the symbiont cell initially appears costly. Nonetheless, these costs are not associated with symbiosis per se but with the inability of autotrophs to keep all the fixed carbon inside the cell, independent of a free-living or host-associated life style. Such costs can be allocated to the symbiosis only if they are enhanced and controlled by the host. Finally, we consider the provision of sulfide and oxygen for chemosynthesis as a byproduct benefit provided by the host through its contracting and expanding behavior as well as by its ciliary movement (**Figure 11**).

Several mechanisms identified in evolutionary theory are crucial for the maintenance of mutualism: (1) partner choice, (2) partner sanctions, (3) and partner fidelity feedback (Bull and Rice, 1991; Noë and Hammerstein, 1994; Johnstone and Bshary, 2002; West et al., 2002a,b; Sachs et al., 2004; Weyl et al., 2010; Archetti et al., 2011). Their importance differs according to the mode of transmission (Ewald, 1987; Douglas, 2010; Sachs et al., 2011). In horizontal transmission, partner choice is crucial for the establishment, during which a cooperative symbiont is selected from the environment in advance of any possible exploitation (Bull and Rice, 1991). In contrast, during vertical transmission, the partner has already been chosen and is transferred to the next generation with high fidelity. Based on our current state of knowledge, this appears to be the case in the *Z. niveum* symbiosis.

Consensus exists on the crucial role of partner fidelity feedback in mutualism with vertical transmission that ensures maintenance after establishment (Douglas, 2010; Sachs et al., 2011). In the *Z. niveum* symbiosis, some of the interactions might be based on partner fidelity feedback (**Figure 11**). The host’s behavior, which supplies the symbionts with chemicals for chemosynthesis (a byproduct benefit), boosts symbiont fitness while increasing host fitness through nourishment. Cessation or decrease of host contraction-expansion behavior and ciliary movement directly negatively affect host fitness. This would also decrease the oxygen supply for the host’s respiration and restrict food uptake by impacting the ciliary movement of microzooids. In addition, the oxygen and sulfide supply fueling chemosynthesis by the symbiont would be diminished, impeding the translocation of organic carbon from the symbiont to the host.

If the symbiont defects by reducing the amount of fixed carbon translocated to the host, then host growth would be reduced, decreasing the host surface available for symbiont colonization. Host growth and symbiont population density are finely tuned, sustaining a monolayer. Accordingly, a defecting and therefore fitter symbiont would overgrow the host unless the latter can sanction the cheater. If, however, the provision of goods to the host is a byproduct of carbon fixation, then the symbiont cannot defect, and partner fidelity feedback would regulate the provision (see Weyl et al., 2010; Archetti et al., 2011).

## CONCLUSION

This review illustrates our current state of knowledge on the *Z. niveum* – *Cand.* Thiobios zoothamnicoli symbiosis. Its extremely wide geographical distribution points to a cosmopolitan symbiosis in tropical to temperate shallow-water environments in which oxic–anoxic interfaces develop on decaying plants or animals. This association is specific for both partners, and the symbiont is permanently associated with the host and transferred vertically to the next host generation. It is obligate for the host, but whether or not it is obligate for the symbiont remains to be determined. Of all thiotrophic symbiosis, this mutualistic association has the highest potential of becoming a model system to study interspecies cooperation and the molecular mechanisms by which host and symbiont initiate the association and interact to persist. It can be cultivated and manipulated, and we recently successfully separated the partners and cultivated the aposymbiotic host (Bright M., Espada-Hinojosa S., Volland J. -M., personal observations). This opens the door to experimentally study the pre- and postinfection mechanisms.

## AUTHOR CONTRIBUTIONS

Monika Bright wrote the manuscript and designed **Figures 3** and **11**, Jean-Marie Volland designed **Figures 1, 7, 8**, and **10**, Ilias Lagkouvardos performed the symbiont phylogenetic analyses and designed **Figure 6**, Salvador Espada-Hinojosa performed the literature search, all authors discussed extensively the content of this review.

## Conflict of Interest Statement

The authors declare that the research was conducted in the absence of any commercial or financial relationships that could be construed as a potential conflict of interest.
